# Diagnostic Phase of Calcium Scoring Scan Applied as the Center of Acquisition Window of Coronary Computed Tomography Angiography Improves Image Quality in Minimal Acquisition Window Scan (Target CTA Mode) Using the Second Generation 320-Row CT

**DOI:** 10.1155/2016/1017851

**Published:** 2016-02-10

**Authors:** Eriko Maeda, Kodai Yamamoto, Shigeaki Kanno, Kenji Ino, Nobuo Tomizawa, Masaaki Akahane, Rumiko Torigoe, Kuni Ohtomo

**Affiliations:** ^1^Department of Radiology, Graduate School of Medicine, University of Tokyo, 7-3-1 Hongo, Bunkyo-ku, Tokyo 113-8655, Japan; ^2^Imaging Center, The University of Tokyo Hospital, 7-3-1 Hongo, Bunkyo-ku, Tokyo 113-8655, Japan; ^3^Department of Radiology, New Tokyo Hospital, 1271 Wanagaya, Matsudo, Chiba 270-2232, Japan; ^4^Department of Radiology, NTT Medical Center Tokyo, 5-9-22 Higashi-Gotanda, Shinagawa-ku, Tokyo 141-8625, Japan; ^5^Toshiba Medical Systems Corporation, Tokyo Metropolitan Regional Office, 1-6 Tsukuda 2-Chome, Chuo-ku, Tokyo 104-0051, Japan

## Abstract

*Objective.* To compare the image quality of coronary computed tomography angiography (CCTA) acquired under two conditions: 75% fixed as the acquisition window center (Group 75%) and the diagnostic phase for calcium scoring scan as the center (CS; Group CS).* Methods.* 320-row cardiac CT with a minimal acquisition window (scanned using “Target CTA” mode) was performed on 81 patients. In Group 75% (*n* = 40), CS was obtained and reconstructed at 75% and the center of the CCTA acquisition window was set at 75%. In Group CS (*n* = 41), CS was obtained at 75% and the diagnostic phase showing minimal artifacts was applied as the center of the CCTA acquisition window. Image quality was evaluated using a four-point scale (4-excellent) and the mean scores were compared between groups.* Results.* The CCTA scan diagnostic phase occurred significantly earlier in CS (75.7 ± 3.2% vs. 73.6 ± 4.5% for Groups 75% and CS, resp.,* p* = 0.013). The mean Group CS image quality score (3.58 ± 0.63) was also higher than that for Group 75% (3.19 ± 0.66,* p* < 0.0001).* Conclusions.* The image quality of CCTA in Target CTA mode was significantly better when the center of acquisition window is adjusted using CS.

## 1. Introduction

Adult coronary computed tomography angiography (CCTA) usually begins with a noncontrast electrocardiogram-gated chest CT called a “calcium scoring (CS) scan” performed after scout scans. CS is used to determine the range of the CCTA scan and to calculate an Agatston score, the counterpart to the calcium score which is obtained using electron beam CT [1]. Because evaluation of the coronary lumen during CCTA is hampered by dense calcification of the coronary artery wall, the Agatston score can be used to select cases with diffuse coronary calcifications, who should not receive further scans due to the likelihood of limited benefit and the risks associated with contrast material and additional radiation exposure [2–6].

Second generation 320-row CT scanners with a rotation speed of 275 ms can scan the whole heart in one rotation, using a minimal acquisition window (“Target CTA”; Toshiba, Tochigi, Japan). This scan mode can be applied for evaluating cases with a heart rate lower than approximately 75 beats per minute (bpm). With Target CTA scans, the center of the acquisition window is set to any integral percentage, and X-ray exposure is limited to only the minimum duration needed to reconstruct the images. Although the acquisition window is set using only one integral, such as 75%, the scan has a short reconstruction window and the diagnostic phase (i.e., the phase showing minimal artifacts) can be searched for within the acquisition window. As an example, use of a Target CTA scan of 75% in a patient with an RR interval of 1000 ms results in an acquisition window of 689–811 ms, with the center of the acquisition window at 750 ms and the width of reconstruction window at 122 ms (note: the acquisition window or the exposure duration always exceeds the reconstruction window). These phase types are searched using “PhaseNavi” cardiac-phase search software (Toshiba, Tochigi, Japan), which automatically searches for the phase that produces the lowest average SD value for all voxels in the volume. However, the results of automated phase searching do not always correspond with the diagnostic phase. Also, the most static phase needs to be visually searched using the same software, if the coronary arteries contain motion artifacts at the point identified by the automated phase search. Compared with other methods, Target CTA is reported to produce low-dose scans together with noninferiority in image quality [7, 8]. The value of 75% is widely used as the center of the Target CTA acquisition window, although this value is empirical [9–13].

CS has been scanned using a 75% Target CTA mode for patients with a heart rate (HR) ≤ 75 bpm, and at 40% for those with an HR > 75 bpm. During the CS Target CTA scan, the reconstruction phase was fixed to 75%, and it was not possible for PhaseNavi software to adjust the reconstruction phase. However, a recent software upgrade (Aquilion ONE ViSION edition version 6.0; Toshiba, Tochigi, Japan) allows for the adjustment of CS scan reconstruction window. We hypothesized that the CS diagnostic phase correlates with that of CCTA and that the image quality of CCTA would improve with adjustment of the center of the CCTA acquisition window using the CS diagnostic phase as compared to using a fixed percentage value.

Therefore the aim of this study was to determine potential correlation between CS and CCTA scan diagnostic phases and to compare the CCTA image quality with the use of 75% (Group 75%) versus the CS diagnostic phase (Group CS) as the center of the acquisition window.

## 2. Materials and Methods

This study which was conducted at a single research center was approved by the local ethics committee. Because of this study's retrospective design, the requirement for informed consent prior to study participation was waived.

### 2.1. Patients

The Target CTA scan was applied to the patients with sinus rhythm and an HR ≤ 75 bpm. For patients with arrhythmias, a different acquisition program had to be applied in order to run an arrhythmia exclusion program. Thus patients with sinus rhythm and an HR ≤ 75 bpm were included in our study.

We retrospectively reviewed the records of 162 consecutive patients who underwent CCTA between October 2013 and February 2014. In December 2013, we started to adjust the center of the CCTA acquisition window for Target CTA using the CS diagnostic phase. Single volume Target CTA mode scanning was not used for 81 patients because of the following reasons: nonsinus rhythm (*n* = 13); single beat scan with long acquisition window because of heart rate fluctuation (*n* = 17); multiple heart beat acquisition (*n* = 23); wide-volume scanning performed to evaluate bypass grafts or aorta (*n* = 16); ventricular evaluation prior to catheter ablation (*n* = 3); or irregular protocol for evaluation of complex cardiac anomaly (*n* = 9). The final study group included 81 patients (Group 75%, *n* = 41; Group CS, *n* = 40) who were scanned because of known or suspected coronary artery disease with chest pain and/or dyspnea, or abnormal electrocardiogram, echocardiogram, or treadmill results.

### 2.2. CT Data Acquisition

All patients underwent CT angiography performed using second generation 320-detector row CT for all enrolled patients and prospective electrocardiogram-gated scans were performed in one heartbeat. The scanning parameters were as follows: detector configuration, 320 × 0.5 mm; gantry rotation time 275 ms; and tube potential, 120 kVp. The tube current was set at 150 mA for calcium scoring scan and from 250 mA to 760 mA for CCTA depending on patient body weight. The mean effective dose was derived from the dose length product multiplied by a conversion coefficient for the chest (*κ* = 0.014 mSv × mGy^−1^ × cm^−1^) [14]. The scan length ranged from 12 to 16 cm depending on the size of the heart.

For CS, the center of the acquisition window was set at 75% throughout the period. Until December 2013, the CS reconstruction phase was not adjustable. After December 2013, the reconstruction phase became adjustable, allowing the diagnostic phase with minimal artifacts to be determined at the CT console using PhaseNavi software. The reconstructed slice thickness was 1.0 mm with a 1.0 mm increment. Images were reconstructed using a “medium soft tissue” kernel (FC04). We routinely use this low-pass kernel for cardiac CT because it reduces beam hardening artifacts originating from the vertebra and the aorta.

For CCTA, the center of the acquisition window was empirically fixed at 75% of the RR interval until November 2013 (Group 75%). After December 2013, the center of acquisition window was set at the CS diagnostic phase value (Group CS). For CCTA scans in both groups, the phase with minimum artifacts was determined at the CT console using PhaseNavi software. Half cycle reconstruction was performed for all patients, meaning that there was a full cycle of X-ray exposure but only a half cycle of data was used for reconstruction. The reconstructed slice thickness was 0.50 mm with an increment of 0.25 mm. Images were reconstructed using a “medium soft tissue” kernel (FC04) with Adaptive Iterative Dose Reduction in 3D (AIDR-3D) strong and symmetric cone beam reconstruction [15, 16]. Images were transferred to a workstation (ZIO Station System; Ziosoft, Tokyo, Japan) for processing.

Patients received 22.2 mg I/kg/s of iopamidol 370 mg I/mL (Iopamiron 370; Bayer, Osaka, Japan). Contrast medium was injected for 10 sec and then a 50 : 50 mixed contrast medium and saline for 4 sec, followed by a 30 mL saline flush. Bolus tracking in the ascending aorta was performed using a double threshold of 100 and 260 Hounsfield Units (HU). Patients were assigned to breathe in and hold their breath after the first threshold. The scan started just after the second threshold.

Nineteen patients were being treated with an oral *β*-blocker (e.g., bisoprolol and carvedilol) as a part of their baseline medication. An oral *β*-blocker (20–40 mg of metoprolol) was administered to 18 patients with HR higher than >65 bpm. The patients were told to take the medicine 2 hours prior to CT angiography. Landiolol (Corebeta; Ono Pharmaceutical, Osaka, Japan) was administered intravenously at 0.125 mg/kg when a patient's HR was over 75 bpm during the time between the calcium scoring scan and CCTA. Patients underwent CCTA 4–7 min after injection (*n* = 11). No patient had any contraindication preventing *β*-blocker use, and no *β*-blocker side effects were observed or reported. All patients received 2.5 mg sublingual isosorbide dinitrate (Nitorol; Eisai, Tokyo, Japan) before imaging.

### 2.3. Subjective Image Analysis

Subjective image quality was rated by Kodai Yamamoto and Eriko Maeda, two cardiovascular radiologists with 5 and 11 years of experience, respectively. Both were blinded from the details of the CT data sets, provided in a randomized order, and clinical information. The Society of Cardiovascular Computed Tomography 18-segment classification was applied for the analysis of coronary angiography data [17]. Image quality was graded on a per-segment level, and a study was deemed diagnostic when every anatomically present segment (≥1.5 mm) could be assessed for the presence of atherosclerosis and severity of stenosis. The results were scored according to a four-point scale as previously described: 4, excellent, no artifact; 3, good, mild artifact; 2, acceptable, moderate artifact present, but images still interpretable; 1, unable to evaluate, severe artifact making interpretation impossible [18]. When scores differed between the two readers, the final score was determined by review and consensus.

### 2.4. Objective Image Analysis

**Figure 1:**
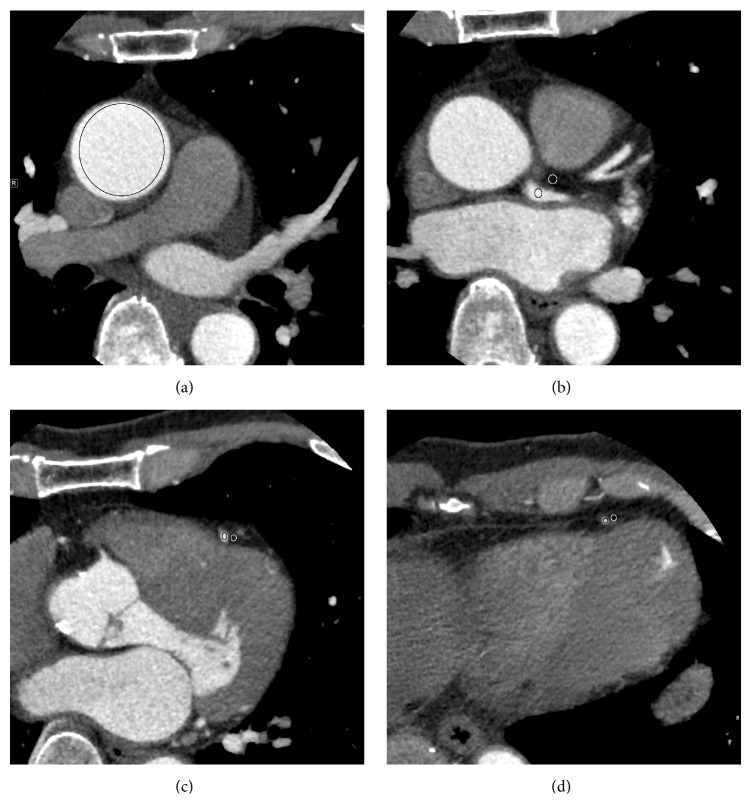
Examples of regions of interest (ROIs) drawn on a cross-sectional image, at the proximal ascending aorta; the proximal, middle, and distal segments of the left anterior descending artery. Black circle shows ROIs drawn inside the lumens of the arteries, while white circle shows ROIs drawn in the nearby connective tissue to calculate contrast-to-noise ratio.

Regions of interest (ROIs) were drawn on a cross-sectional image, at the proximal ascending aorta; the proximal, middle, and distal segments of the right coronary; the left anterior descending artery; and the left circumflex artery. The average CT number (in HU) and noise were recorded for each segment using a circular ROI. The ROI was made as large as possible while carefully avoiding inclusion of the vessel wall to prevent partial volume effects (Figure 1). An ROI was placed immediately next to the vessel contour on an axial image and the average CT number was recorded. The overall signal-to-noise ratio was defined as the average standard deviation of the circular ROI placed at the ascending aorta. The SNR of each coronary vessel was defined as the average standard deviation of the circular ROI placed at the proximal, middle, and distal segments of the vessel. The overall contrast-to-noise ratio (CNR) was calculated as the difference in the CT number between the ascending aortic lumen and nearby connective tissue divided by the overall image noise. For each coronary vessel, CNR was defined as the average CNR of the circular ROI placed at the proximal, middle, and distal segments of the vessel. We expected that the ascending aorta SNR and CNR would not change between the groups, because aortic image noise is unlikely to be related to the motion of the coronary arteries. Therefore we calculated SNR and CNR at the ascending aorta as a control.

### 2.5. Statistical Analysis

A power analysis was performed to determine the minimal cohort size required using G^*∗*^power version 3.1.9.2. (Universitat Düsseldorf, Düsseldorf, Germany). Our hypothesis was that per-segment subjective image quality would improve in Group CS. To detect a difference of 0.1 in subjective image quality score, the minimum sample size was determined to be a total of 527 segments (approximately 30 patients) at 0.90 power. Sample size calculations were based on a type-2 error (*α* = ) of 0.05 [19].

The minimal acquisition window scans (Target CTA mode) for October 2013 were reviewed (*n* = 20) and the reconstruction window was calculated from the console information. The reconstruction window was invariably proven to be 122 ms. To know the percentage of patients in Group CS whose best reconstruction phase would not have been included in the scan if the fixed 75% scan was applied, we compared the actual exposure time as well as “virtual” 75% exposure {i.e., [RR  interval  (ms) × 0.75] ± 61 ms}. The correlation between the CS and CCTA scan diagnostic phases was calculated using Spearman's correlation coefficient analysis.

All statistical analyses were performed using JMP software (version 10; SAS, Cary, NC, USA). Quantitative variables were expressed as the mean ± standard deviation and group differences were tested by Student's *t*-test. Categorical values were expressed as the number (percentage) and were compared using Fisher's exact test or the chi-squared test. Statistical significance was accepted when *p* < 0.05.

## 3. Results

**Figure 2:**
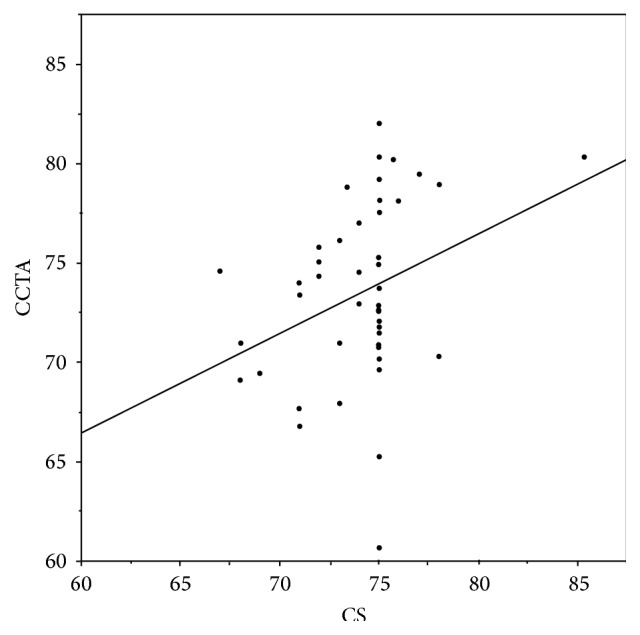
Scatter plot comparing the calcium scoring (CS) scan and coronary computed tomography angiography (CCTA) diagnostic phases for each patient. The line represents predictive formula for the CCTA diagnostic phase, which is CCTA diagnostic phase = 36.3 + 0.5 × CS diagnostic phase.

**Figure 3:**
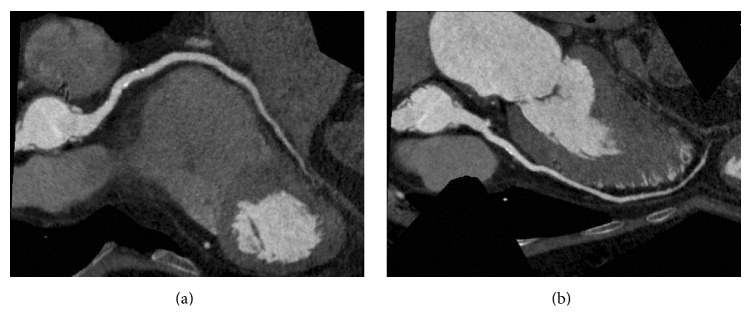
Presurgical screening coronary computed tomography angiography (RR interval = 1114 ms) performed in Target CTA mode on a 61-year-old male with a history of aortic valve replacement. Curved multiplanar reconstruction images show the right coronary (a) and left anterior descending arteries (b) with minimal motion artifacts. The diagnostic phase was 68% for calcium scoring scan and 69.1% for coronary computed tomography angiography. The virtual window of 75% exposure was calculated as 70.0–80.4%, which does not include the CCTA diagnostic phase.

There was no group difference in patient demographics and scanning parameters (Tables 1 and 2). The mean best reconstruction phase (%) for the CCTA scan was significantly earlier for Group CS, although quite widely variable range was observed among the diagnostic phases (Table 3, Figure 2). For eight patients in Group CS, the diagnostic phase occurred outside of the virtual 75% exposure (19.5%) (Figure 3). A significant correlation was detected between CS and CCTA diagnostic phases (Spearman's correlation coefficient 0.351, *p* = 0.02, *R* ^2^ = 0.113).

Among the 1458 total segments captured for each group (18 segments in 81 patients), 137 segments were not evaluable because the segment was absent or too small (60 and 77 segments for Group 75% and Group CS, resp.). The subjective image quality scores were significantly better in Group CS, both for overall and for branch specific analyses (Table 4). Branch specific analyses of objective image quality scores were also higher in Group CS (Table 5). Interobserver agreement on subjective image quality was “good” (*κ* = 0.68). When patients with poor interobserver agreement were defined as “difference in subjective score between two graders being 2 or more in more than five segments,” we found that just four patients qualified as having poor interobserver agreement due to either the presence of dense calcification or multiple stents.

## 4. Discussion

This is the first report on the use of the CS diagnostic phase as the center of the CCTA acquisition window for Target CTA mode scanning. Group CS image quality was significantly better than that for Group 75% using both subjective and objective evaluations. The CS and CCTA diagnostic phases were both earlier than the empirically derived 75%, with the diagnostic phases considered to be outside the 75%-centered acquisition window in 19.5% of cases. The premise of this study of correlation between the CS and CTCAG diagnostic phases was also proven. The correlation efficiency between diagnostic phases of CS and CCTA was 0.351 (*p* = 0.02) indicating weak positive correlation [20].

The greatest advantage of adjusting the center of the acquisition window using CS instead of applying a fixed percentage as the center of acquisition window was an improvement in image quality due to individual adjustments made to the center of the acquisition window. The necessity of this adjustment is based on the wide individual variation in the diagnostic phase and the significant correlation between CS and CCTA scan diagnostic phases. The major disadvantage of this method is the increased workload during the scan as the method requires several additional steps, as compared with using a fixed percentage. CS images need to be reconstructed with a narrower field of view, the diagnostic phase must be identified on multiple planes using cardiac-phase search software and must be reconstructed using the searched phase. This entire sequence of actions needs to be completed in timely fashion (i.e., before the CCTA scan) and must be repeated for certain “difficult” cases. Therefore, the phase search usually requires the input of a radiologist or a technologist in addition to the scanning technologist.

There is a worldwide trend to reduce radiation exposure during cardiac CT. Indeed, radiologists should make their best effort to achieve “as low as reasonable achievable (ALARA)” radiation exposure during every examination. Target CTA scan is a product of the response to this mandate. However, the present study showed wide individual variation in cardiac CT diagnostic phase. Radiologists should tailor the center of the acquisition window during Target CTA scanning or set the acquisition window wider than the narrowest setting to help achieve this goal. For instance, Steigner et al. suggest 72–81% acquisition window has a good probability of including the diagnostic phase for 95% coronary arteries [21]. If Target CTA is to be used without tailoring the center of acquisition window using CS, the accompanying physician or technologist should at least look for motion artifacts on the CS scan before deciding to use Target CTA for CCTA scanning. If coronary arteries on the CS images contain motion artifacts, setting a wider acquisition window than the Target CTA (e.g., 70–80%) raises the probability of obtaining better CCTA images without motion artifacts.

There are some limitations to our study. The heart usually straddles two volumes in CS, because CS needs to cover an area wider than the heart. First, because the RR intervals differ between those volumes, the reconstruction window becomes narrow when there is a large difference in the RR intervals. When this difference is too big, the reconstruction requires an artificial adjustment of the position of one of the R waves. In this study, cases that required such adjustment were considered to be arrhythmic and were excluded, because a longer CCTA acquisition window was applied to such cases. For the second limitation, the center of the CS acquisition window was fixed to 75%. This means that if the diagnostic phase existed at an extreme such as 55% or 92%, it would not be possible to include this phase, even when the center of the acquisition window was adjusted using CS. In fact, with some of our cases, the CS diagnostic phase was located at the earliest pole of the scan. Likewise, the best CCTA reconstruction phase was also found at the earliest pole. With these cases, there is likely to be and even better phase that occurred even earlier. The third study limitation was that many cases exhibited a gap between the CS and CCTA diagnostic phases, as was expected based on the correlation coefficient. In cases with large gaps, an even better phase may exist beyond the CCTA acquisition window. The fourth limitation pertains to the limited sample size because the average Group CS diagnostic phases and the percentage of patients that had values outside of the 75% fixed scan acquisition window were derived from a small number of patients. This point could be addressed by repeating the investigation in a larger population. In addition, further studies should be performed to determine whether the method described herein is also effective with other CT systems, such as 256-row CT or dual-source CT.

## 5. Conclusions

This study found that CS and CCTA diagnostic phases were significantly correlated, with average diagnostic phase of 73.9% and 73.6%, respectively, although the phases showed substantial interindividual variation. The CCTA scan image quality using Target CTA mode was significantly better when the center of the acquisition window was adjusted using CS, compared with that using a fixed percentage.

## Figures and Tables

**Table 1:** Patient demographics.

Parameter	Group 75%	Group CS	*p* value
Number of patients	40	41	
Male/female	28/12	21/20	0.14
Age (years)	66.8 ± 11.8	66.6 ± 9.9	0.94
Body weight (kg)	64.1 ± 14.8	60.7 ± 11.5	0.26
Body mass index (kg/m^2^)	24.4 ± 3.8	24.2 ± 3.9	0.83
Beta-blocker^+^	15 (38)	19 (46)	0.28
Heart rate (bpm)	58.9 ± 6.5	57.7 ± 7.0	0.44
Coronary risk factor^+^			
Hypertension	21 (53)	26 (63)	0.18
Diabetes mellitus	10 (25)	12 (29)	0.43
Dyslipidemia	21 (53)	25 (61)	0.29
Smoking	17 (43)	17 (41)	0.55
Family history	3 (8)	5 (12)	0.37

^+^Data represent the number of patients (percentage).

**Table 2:** Scanning parameters.

Parameter	Group 75%	Group CS	*p* value
Contrast medium (mL)	45.5 ± 9.4	42.8 ± 8.0	0.17
Injection rate (mL/sec)	3.8 ± 0.8	3.6 ± 0.6	0.13
Tube current (mA)	397 ± 125	354 ± 92	0.08
Scan length (cm)	13.1 ± 1.3	12.9 ± 1.2	0.47
Effective dose (mSv)	1.87 ± 0.75	1.70 ± 0.66	0.28

**Table 3:** Comparison of reconstruction phases between both reconstruction methods.

	Group 75%	Group CS	*p* value
CS scan (%)			
Average	75 (unadjustable)	73.9 ± 3.0	N/A
Range	N/A	67.0–85.3	
CCTA scan (%)			
Average	75.7 ± 3.2	73.6 ± 4.5	0.013^*∗*^
Range	70.2–81.2	60.8–82.0	

^*∗*^Statistically significant.

**Table 4:** Subjective image quality.

	Group 75%	Group CS	*p* value
Overall	3.20 ± 0.66	3.58 ± 0.63	<0.0001^*∗*^
RCA	3.18 ± 0.65	3.63 ± 0.60	<0.0001^*∗*^
LMT + LAD + HL	3.23 ± 0.66	3.58 ± 0.62	<0.0001^*∗*^
LCX	3.17 ± 0.67	3.55 ± 0.67	<0.0001^*∗*^

^*∗*^Statistically significant.

RCA: right coronary artery (#1–4 and #16).

LMT + LAD + HL: left main trunk, left anterior descending, and high lateral branch (#5–10 and #17).

LCX: left circumflex artery (#11–15 and #18).

**Table 5:** Objective image quality.

	Group 75%	Group CS	*p* value
Signal-to-noise ratio			
Overall	21.5 ± 2.0	21.5 ± 2.1	0.98
RCA	20.6 ± 4.6	13.4 ± 3.1	<0.0001^*∗*^
LAD	21.1 ± 4.3	14.8 ± 2.8	<0.0001^*∗*^
LCX	19.7 ± 3.3	16.1 ± 3.0	0.0038^*∗*^
Contrast-to-noise ratio			
Overall	25.2 ± 5.7	23.1 ± 4.0	0.24
RCA	27.5 ± 5.4	40.7 ± 12.5	0.0023^*∗*^
LAD	26.9 ± 7.0	35.6 ± 9.9	0.015^*∗*^
LCX	26.2 ± 6.5	31.4 ± 9.4	0.112^*∗*^

^*∗*^Statistically significant.
